# Toll-like receptor 2 modulates age-associated insulitis and fibrotic remodeling of Langerhans islets

**DOI:** 10.1186/s12979-026-00598-0

**Published:** 2026-09-12

**Authors:** Julia Jelleschitz, Annette Brandt, Klara Brehm, Vanessa Schnell, Tobias Jung, Ina Bergheim, Annika Höhn

**Affiliations:** 1 Department of Molecular Toxicology, German Institute of Human Nutrition, Potsdam-Rehbruecke (DIfE), Arthur-Scheunert-Allee 114-116, Nuthetal, 14558 Germany; 2 Department of Nutritional Sciences, Molecular Nutritional Science, University of Vienna, Vienna, Austria; 3 German Center for Diabetes Research (DZD), München-Neuherberg, Germany

**Keywords:** Aging, TLR2, Langerhans islets, Insulitis, Immune cells, Fibrosis

## Abstract

**Background:**

The steadily increasing aging population highlights the importance of maintaining health at an advanced age. Metabolic diseases are among the most prevalent age-related disorders, and the Langerhans islets, critical regulators of glucose homeostasis, are particularly susceptible to disturbed insulin secretion and functional decline. Multiple stimuli contribute to islet dysfunction during aging, including bacterial products that increase with age and can activate Toll-like receptor (TLR) signaling. TLR2, which recognizes a broad spectrum of microbial ligands including components from Gram-positive bacteria, has been implicated in type 2 diabetes; however, its role in β-cell function during aging remains unclear. Accordingly, we hypothesize that TLR2 signaling contributes to inflammation and remodeling during aging in Langerhans islets.

**Results:**

To determine the role of TLR2 in islet aging, we compared young and old male TLR2-deficient mice with age-matched C57BL/6J controls. Old TLR2^−/−^ mice showed an altered ex vivo glucose-stimulated insulin secretory response, together with attenuated age-related islet hypertrophy and β-cell mass expansion. Immunohistochemical analysis further revealed that this was accompanied by decreased intra-islet immune cell accumulation, in particular macrophages, but also reduced fibrosis. In a complementary approach, 17-month-old C57BL/6J mice with elevated levels of TLR2 ligands were pharmacologically treated with the TLR2 inhibitor ortho-vanillin (60 mg/kg bodyweight) for four months. This intervention attenuated insulitis by reducing intra-islet leukocyte accumulation, particularly macrophage infiltration, and significantly reduced islet fibrosis, as indicated by decreased collagen and αSMA staining. Since mice at the investigated age-time points do not show metabolic impairments of glucose handling, these histological improvements were not yet reflected by improved glucose tolerance.

**Conclusions:**

Overall, our results suggest that TLR2 signaling contributes to immune cell accumulation and fibrotic remodeling during aging, linking inflammation to pancreatic islet aging in mice. These findings identify TLR2 as a potential mediator of age-related islet dysfunction and a candidate target for further investigation.

**Supplementary Information:**

The online version contains supplementary material available at https://doi.org/10.1186/s12979-026-00598-0.

## Background

Rising life expectancy over recent decades has led to a steady increase in the proportion of older adults worldwide [1]. However, healthy life span is not increasing at the same rate [2], but instead the prevalence of age-related diseases increases, driven by multiple factors including progressive decline in tissue function during aging. Contributing factors such as oxidative stress, inflammatory mediators and cellular senescence closely interact and promote chronic low-grade inflammation, thereby increasing susceptibility to metabolic diseases such as metabolic syndrome, type 2 diabetes or cardiovascular diseases [3–5]. Maintaining proper function of metabolic organs, including the pancreas, is therefore crucial during aging. Pancreatic Langerhans islets are essential for maintaining glucose homeostasis [6], and insulin-producing β-cells as post-mitotic, long-lived cells with minimal turnover are particularly susceptible to metabolic and inflammatory stressors [7–9]. Together, this highlights the need to identify strategies that preserve β-cell function and survival into advanced age.

Indeed, studies have shown that aged Langerhans islets show marked signs of inflammation, immune cell accumulation, oxidative stress and fibrotic remodeling [10–14]. These changes may involve activation of inflammatory signaling cascades by toll-like receptors (TLRs), which are highly expressed by immune cells and recognize damage-associated patterns [15, 16]. With aging, intestinal barrier dysfunction has been linked to increased systemic exposure to bacterial products, including TLR2 and TLR4 ligands [17]. While TLR4 recognizes lipopolysaccharides from Gram-negative bacteria, its activation in aging has been associated with enhanced inflammation and fibrosis in various organs such as the liver [18, 19], and, as recently shown by us, is also critical in Langerhans islets [20]. Emerging evidence further suggests also a pivotal role for TLR2 activation in age-related liver dysfunction and cellular senescence [21]. Because TLR2 can signal as a homodimer (TLR2/2) or as a heterodimer with TLR1 (TLR2/1) or TLR6 (TLR2/6), and more rarely with TLR10 (TLR2/10), this receptor recognizes a broad spectrum of ligands and may exert context-dependent pro- or anti-inflammatory effects [22]. TLR2 activation can induce formation of reactive oxygen species and pro-inflammatory mediators that promote metabolic dysfunction, and TLR2-deficient mice have been reported to be protected from insulin resistance, potentially through enhanced hepatic insulin action [23, 24]. However, little is known about the effect of TLR2 on islet function and age-related islet remodeling.

This study investigates the contribution of TLR2 signaling to age-associated histological remodeling of pancreatic islets at an age when glucose handling remains preserved, focusing on changes that may precede overt metabolic dysfunction. In two complementary mouse studies genetic and pharmacological inhibition of TLR2 were examined. First, young and old C57BL/6J mice were compared to age-matched TLR2^−/−^ mice. Second, aged C57BL/6J mice, were treated pharmacologically with the TLR2 inhibitor ortho-vanillin (oV) for four months starting at an age when bacterial-derived ligands start to be elevated. Glucose tolerance was assessed while focusing on immune cell infiltration and subtypes in pancreatic tissue sections.

## Materials and methods

### Animal procedures and study design

#### General animal care and ethics

All experiments complied with the European Directive 2010/63/EU, the National Institutes of Health (NIH) guidelines for the care and use of laboratory animals, and site-specific institutional regulations described below. Animals and samples were obtained at two sites. At the German Institute of Human Nutrition Potsdam-Rehbruecke (DIfE), mice were used exclusively as untreated tissue donors for organ collection and pancreatic islet isolation. Before euthanasia, they were maintained under standard husbandry conditions according Sect. 11 of the German Animal Welfare Act (TierSchG) and were not subjected to any experimental intervention, treatment, or functional testing. Animals were euthanized solely to obtain organs and tissues for scientific use. This procedure was covered by Sect. 4 (3) TierSchG and, according to Sect. 7 (2), sentence 3, no. 1 TierSchG, is not classified as an animal experiment. At the University of Vienna, Austria, all procedures were approved by the institutional ethics and animal welfare committee and the Federal Ministry of Education, Science and Research (BMBWF, 2021 − 0.464.120, 2022 − 0.527.442).

Mice were maintained under specific pathogen-free conditions in a controlled environment (20 ± 2 °C, 12:12 h light/dark cycle). At the University of Vienna, lights were on from 7:00 a.m. to 7:00 p.m., while at the MRL of the DIfE, they were on from 5:30 a.m. to 5:30 p.m. Animals had ad libitum access to standard chow (Ssniff, Soest, Germany) and water, unless otherwise specified.

At DIfE, animals were euthanized under anesthesia by acute isoflurane inhalation followed by cervical dislocation. At the University of Vienna, animals were euthanized under anesthesia by intraperitoneal (i.p.) ketamine (100 mg/kg body weight (BW)) plus xylazine (16 mg/kg BW) followed by cervical dislocation and collection of blood and tissue. Langerhans islets were isolated following cervical dislocation.

#### Aging cohort of full body TLR2 knockout and controls

Male C57BL/6J (BL6,#000664, The Jackson Laboratory, USA) and TLR2 Knockout (TLR2^−/−^, B6.129-Tlr2tm1Kir/J, #:004650, The Jackson Laboratory, USA), were bred and housed in the SPF animal facility at the University of Vienna in groups under controlled conditions. BL6 and TLR2^−/−^ mice were maintained in separate genotype-specific cages under identical husbandry conditions in the same animal room. The animals were neither littermates nor co-housed. Experiments involving both genotypes were conducted in parallel. Mice had ad libitum access to water and standard chow and were aged to 4 months (young) or 20 months (old). One week prior to sacrifice, a glucose tolerance test (GTT) was performed as detailed below (see Glucose tolerance test Section.). At sacrifice, blood was collected from the portal vein and pancreatic tissue was harvested and fixed in neutral buffered formalin. From a subset of mice, islets of Langerhans were isolated for glucose-stimulated insulin secretion (GSIS) experiments (see Islet isolation and Glucose stimulation of Langerhans islets (GSIS) Sections).

#### Pharmacological inhibition of TLR2

Seventeen-month-old male C57BL/6J mice bred in-house at the University of Vienna, were either treated with TLR2 inhibitor ortho-vanillin (oV, 60 mg/kg BW in drinking water, Sigma Aldrich, Germany) or plain drinking water as control for 4 months. The intervention was initiated at 17 months based on our previous observation of significantly increased circulating TLR2 ligands at this age [21]. Glucose tolerance was measured one week prior to sacrifice (see Glucose tolerance test Section.). At sacrifice, blood was collected from the portal vein, and pancreatic tissue was harvested and fixed in neutral buffered formalin.

#### Mice for ex vivo islet stimulation and TLR2-ligand quantification

Additional young and old C57BL/6J mice bred in-house (Max Rubner Laboratory, DIfE) were used for ex vivo experiments and tissue collection and were euthanized as described in General animal care and ethics Section. Pancreatic tissue or islets of Langerhans were collected. Isolated islets from old C57BL/6J mice were used for ex vivo stimulation with 10 µg/ml lipoteichoic acid (LTA) in the presence of 50 µM oV, and pancreatic tissue from young and old animals was collected for quantification of TLR2 ligands.

### Glucose tolerance test

A GTT was performed in TLR2^−/−^mice as well as in TLR2 inhibitor-treated mice and their respective controls, to assess the metabolic response to glucose. After a 6-hour fast one week before sacrifice, BW and fasting blood glucose were measured from blood collected of the lateral tail vein. Mice then received an i.p. injection of glucose (2 g/kg BW, 20% glucose solution), and blood glucose levels were measured from the lateral tail vein at 15, 30-, 60-, 90-, and 120-minutes post-injection. Data were visualized as blood glucose concentration over the time to evaluate the glucose tolerance curve and glucose concentrations at different time points between the animal groups were compared.

### Islet isolation

For assessment of glucose-stimulated insulin secretion, Langerhans islets were isolated from young and old TLR2^−/−^ and BL6 mice. Additionally, islets isolated from old C57BL/6J mice were stimulated with LTA, a cell-wall component of Gram-positive bacteria and a TLR2 ligand, in the presence or absence of oV. Subsequent gene-expression analyses were performed to assess the direct responsiveness of pancreatic islets to TLR2 stimulation. The isolation of Langerhans islets was performed following a previously described protocol [20]. Briefly, directly after sacrifice, 3 ml Liberase TL solution (Sigma Aldrich, Germany) was injected into the common bile duct, followed by digestion in 2 ml Liberase TL solution at 37 °C for 18 min. After mechanical dissociation of the pancreas, the exocrine part was separated by a Histopaque-Percoll gradient and islets were handpicked under a stereomicroscope. Subsequently, the islets were cultivated overnight at 37 °C in RPMI^+^ media (BIO&SELL, Germany, 2 g/l glucose (11.1 mM), supplemented with L-glutamine, penicillin/Streptomycin, and 10% fetal bovine serum) for further assays.

### Glucose stimulation of Langerhans islets (GSIS)

For GSIS ~ 40 islets per animal from young and old TLR2^−/−^ and BL6 mice were allowed to recover overnight after isolation at 37 °C in RPMI^+^ medium. Following a previously described protocol [10, 12, 20], islets were washed in Krebs-Ringer bicarbonate HEPES buffer (KRBH) containing 0.1% bovine serum albumin (BSA). After equilibration in KRBH, insulin secretion was assessed under low glucose conditions (LG, KRBH + 1mM glucose), followed by high glucose stimulation (HG, KRBH + 25 mM glucose). A stimulation index was calculated as the ratio of HG to LG insulin values measured in the supernatants after stimulation. For total insulin secretion, islets were depolarized with 40 mM potassium chloride (KCl) in presence of 1 mM glucose. Total secreted insulin was calculated as the sum of insulin released into the collected supernatants (LG + HG + KCl) and normalized to islet DNA content.

### Determination of insulin and DNA content

Insulin was determined in murine plasma samples as well as in supernatants collected during GSIS using an ultrasensitive mouse insulin ELISA (ALPCO, USA) according to the manufacturer’s manual. Insulin concentrations in GSIS supernatants were normalized to islet DNA content. Therefore, islets used in GSIS were lysed, sonicated and DNA was quantified using the PicoGreen dsDNA assay (Thermo Fisher Scientific, Germany) according to the manufacturer’s manual. Fluorescence was measured at an excitation wavelength of 450 nm and an emission wavelength of 520 nm, and DNA content of islets was calculated from a standard curve.

### Immunohistochemistry and immunofluorescence of paraffin embedded pancreatic tissue sections

Immunohistochemistry (DAB): Paraffin-embedded pancreatic tissue was sectioned at 2 μm and stained with DAB Chromogen (Agilent, Germany) with hematoxylin counterstaining as previously described [10, 20]. Briefly, sections were deparaffinized, antigen retrieval in citric buffer (pH 6.0) was performed and sections were washed in phosphate-buffered saline (PBS), followed by permeabilization in PBS + 0.2% Triton-X-100 and blocking buffer incubation (DAKO antibody diluent, Agilent, USA + 10% normal goat serum). For the analysis of β-cell mass, insulin (1:40 000 diluted, ab181547, Abcam, United Kingdom) was stained in 3 independent sectional planes. For the calculation of the β-cell mass, insulin^+^ area and total pancreas area were determined by threshold-based analysis in Zen version 2.6/3.5. The ratio of insulin/pancreas was averaged across all sectional planes per animal and multiplied by pancreas weight (β-cell mass = (Insulin^+^ area/total pancreas area) * pancreas weight). Macrophage accumulation in the islets was determined via CD68 (1:100, ab125212, Abcam, United Kingdom) staining. For the analysis, CD68 positive cells were counted in the islet, about 10–20 islets per animal were analyzed.

Immunofluorescence: For detailed analysis of Langerhans islets additional immunofluorescence staining was conducted as previously described [10, 12, 20, 25]. In brief, 2 µM thick pancreatic tissue sections were deparaffinized and antigen retrieval in citrate buffer (pH 6.0) or EDTA buffer (pH 9.0; only for CD3 staining) was implemented, followed by washing steps and blocking for 1 h at room temperature (RT). Subsequently, overnight primary antibody incubation at 4° was performed, followed by washing steps and secondary antibody incubation (anti-mouse 488 A-11029, anti-mouse 633 A-21052, anti-rabbit 633 A-21071, anti-rabbit 488 A-11008, anti-rat 546 A-11081 or anti-rat 633 A-21094, Invitrogen, USA) including DAPI for 1 h at RT and mounting. For the analysis of islet composition and mean islet size, insulin (1:100, MAB1417, Novus Biologicals, Germany), glucagon (1:200, ab10988, Abcam, United Kingdom) and somatostatin (1:200, ab111912, Abcam, United Kingdom) were co-stained and areas were quantified by threshold-based analysis in Zen 3.5. To identify activated pancreatic stellate cells, α-smooth muscle actin, (αSMA 1:500, #19245, Cell Signaling, Germany) was co-stained with insulin (1:200, #8138, Cell Signaling, Germany). Threshold based areas of αSMA and insulin were analyzed in Image J or Zen 3.5, and the αSMA/insulin area ratio was calculated. To identify accumulating immune cells in Langerhans islets, CD45 (1:50, #559864, Biosciences, Germany) and CD3 (1:50, ab11089, Abcam, United Kingdom) were stained and positive cells were counted per islet; ~10–20 islets per animal were analyzed. Defined image-analysis settings and cut-off values for αSMA, insulin, and immune-cell positivity are reported in Supplementary Table S1.

Imaging: Slides were scanned using a MIRAX scanner or AxioScan Z.1/ Axioscan 7 (Zeiss, Germany) using a 5x magnitude for coarse focus and a 20x/0.8 objective for high-resolution scanning. Image analysis was performed in Zen 2.6/3.5 or Image J (αSMA).

### Histopathological scoring of insulitis and fibrosis

Immune cell infiltration of Langerhans islets (insulitis), and islet fibrosis were assessed using established scoring systems based on previously published criteria (see Supplementary Fig. S1) [10, 26–29]. For insulitis evaluation, pancreatic sections were immunohistochemically stained for insulin to delineate Langerhans islets and enable assessment of infiltrating immune cells. Scoring to classify immune cell infiltration patterns of four grades, was performed by two independent investigators in a blinded manner across three sectional planes per pancreas sample. To visualize collagen and classify fibrosis grades, pancreatic tissue sections were stained for Masson’s Trichrome. Islet fibrosis was graded on a five-stage scale by two blinded investigators. Agreement between the two independent investigators was assessed using weighted Cohen’s κ and is reported in Supplementary Table S2.

### TLR2 ligands in young and old pancreatic tissue

TLR2 ligands in pancreatic tissue samples from young and old C57BL/6 were determined with a commercially available SEAP-reporter assay in transfected HEK293 cells (Invivogen, USA) as described before [20, 30]. Briefly, snap-frozen pancreatic tissue was homogenized in pyrogen-free water, sonicated for 1 min, and applied to the reporter cells. Optical density was measured at 655 nm and normalized to protein content of each sample.

### In vitro culture of Langerhans islets and stimulation

For recovery after isolation, islets from old mice were incubated overnight in RPMI^+^ medium. The next day, islets were pooled and transferred into a 24-well plate (*n* = 3 biological replicates, 200 islets per condition, four conditions). The following conditions were tested: (i) vehicle control (PBS / 0.9% NaCl), (ii) oV (50 µM;100 mM stock in 0.9% NaCl, Sigma Aldrich, Germany), (iii) LTA (10 µg/ml; 5 mg/ml stock solution in PBS, from *Bacillus subtilis*, Sigma Aldrich, Germany), and (iv) oV pre-treatment (50 µM) followed by LTA stimulation (10 µg/ml). Equivalent amounts of 0.9% NaCl and PBS were included in all conditions as vehicles. A 2-hour pre-treatment with oV or 0.9% NaCl was followed by 4 h stimulation with LTA or PBS, respectively. Subsequently islets were handpicked, washed three times with PBS and snap-frozen for mRNA isolation and gene expression analysis. Since both controls (vehicle only or oV) showed similar gene expression, only the vehicle control is shown in the results.

### mRNA Isolation and quantitative polymerase chain reaction (qPCR)

Isolation of total mRNA from islets was performed using the Dynabeads™ mRNA DIRECT™ Kit (Invitrogen, USA) according to the manufacturer’s protocol. Samples were reverse-transcribed into cDNA and qPCR was conducted as described before [20], with cDNA diluted 1:3 in nuclease-free water. Primers were obtained from Sigma Aldrich, Germany and forward and reverse primers were used at a final concentration of 1.25 µM. The following primer pairs were used: tumor necrosis factor alpha (TNFα, forward: 5’-CCACGTCGTAGCAAACCACC-3’, reverse: 5’-TACAACCCATCGGCTGGCAC-3’), nitric oxide synthase, inducible (Nos2/iNOS, forward: 5’-CAGCGCTACAACATCCTGGAGG-3’, reverse: 5’- GGACCAGCCAAATCCAGTCTGC-3’), interleukin 1-beta (IL-1ß, forward: 5’-TTGAAGAAGAGCCCATCCTCTGTG-3’, reverse: 5’-TTGTTCATCTCGGAGCCTGTAGTG-3’), insulin 2 (Ins2, forward: 5’-TTTGCTATCCTCAACCCAGCC-3’, reverse: 5’- CAGGTGGGAACCACAAAGGT-3’), and interleukin 6 (IL-6, forward: 5’-TCTCTGCAAGAGACTTCCATCCA-3’, reverse: 5’- GTCTGTTGGGAGTGGTATCCTCTG-3’). As housekeeping genes β-Actin (forward: 5’-CACTGCCGCATCCTCTTCCT-3’, reverse: 5’-GATTCCATACCCAAGAAGGAAGGC-3’) and hypoxanthine-guanine phosphoribosyl transferase 1 (HPRT 1, forward: 5’-GCAGTCCCAGCGTCGTG-3’, reverse: 5’-GGCCTCCCATCTCCTTCAT-3’) were taken. Relative gene expression was calculated by the Bio-Rad CFX Maestro 2.3 Software and normalized to the housekeeping genes (based on ΔΔCt method). Due to low gene expression of TLR2 in islets, a specific TaqMan^®^ Gene Expression Assay (msTLR-2_Mm00442346_m1, Thermo Fisher Scientific, Germany) was used to enhance the signal and performed according to the manufacturer’s protocol with an annealing temperature of 60 °C. TLR2 expression was normalized to the both reference genes using the ΔΔCt method and is presented as logΔΔCt in the results.

### Statistical analysis

Statistical analyses were performed with GraphPad Prism Version 10.6.1. For sample groups with *n* > 3, outliers were assessed using the Grubb’s test. For comparison between two groups either unpaired Student’s *t*-test (data normally distributed) or Mann-Whitney *U* test (data non-parametrically distributed) were conducted. For comparison between three groups, an ordinary one-way ANOVA with post-hoc Tukey’s-multiple comparisons test was performed For grouped data, two-way ANOVA followed by uncorrected Fisher’s LSD testing was used for a limited number of biologically defined comparisons addressing age effects within genotypes and genotype effects within age groups. Glucose tolerance curves were analyzed by Two-Way ANOVA (Tukey’s or Šídák’s multiple comparison test) comparing blood glucose values of different time points between groups. All values are presented as mean ± standard deviation (SD) unless otherwise stated. For ordinal data such as insulitis and fibrosis scoring only the mean value is reported. Results with a p-value < 0.05 were considered as statistically significant, while directional differences with p-values between 0.05 and 0.10 are reported as trends but are explicitly considered not statistically significant.

## Results

### Genetic TLR2 deletion enhances glucose stimulated insulin secretion in old mice

**Fig. 1:**
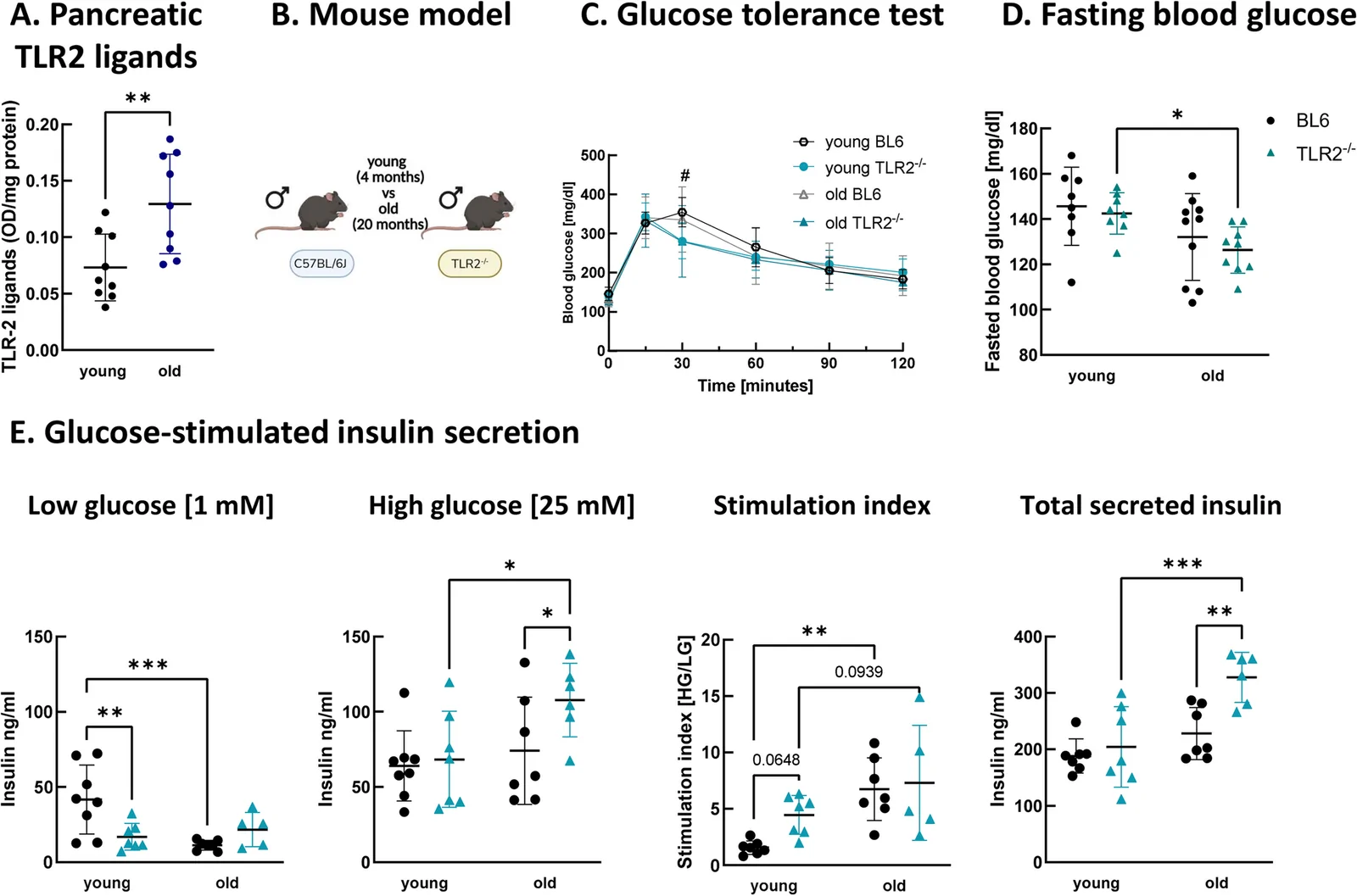
Effect of genetic deletion of TLR2 on glucose metabolism and insulin secretion in young and old mice. **A** Assessment of TLR2 ligands in pancreatic tissue of young and old C57BL/6J mice; *n* = 10; unpaired Student’s *t*-test; ** *p* < 0.01 **B** Design of the mouse model to compare full-body TLR2 knockout (TLR2^−/−^) mice to C57BL/6 (BL6) mice; graphic created in BioRender. **C** Glucose tolerance test of young and old TLR2^−/−^ and BL6 mice; *n* = 8–10; Two-Way ANOVA with Tukey’s multiple comparisons test; #: significant difference (*p* = 0.0162) between young TLR2^−/−^ mice and young BL6 mice, and a trend for a difference between old TLR2^−/−^ and old BL6 mice (*p* = 0.0590) 30 min after glucose injection. **D** Fasting blood glucose levels of young and old TLR2^−/−^ and BL6 mice; *n* = 8–10; Two-Way ANOVA with Uncorrected Fisher’s LSD; *p < 0.05. **E** Glucose-stimulated insulin secretion of isolated Langerhans islets of young and old TLR2^−/−^ and BL6 mice. Concentration of secreted insulin under low glucose (LG) and high glucose (HG) stimulation with stimulation index (HG/LG) and calculation of total insulin secretion (LG + HG + KCl); *n* = 5–8; Two-Way ANOVA with Uncorrected Fisher’s LSD; *p < 0.05; **p < 0.01; ***p < 0.001. All Data are represented as mean ± SD. TLR = toll-like receptor

As we have previously demonstrated, aging Langerhans islets are affected by various remodeling changes, including increased immune cell infiltration and fibrosis [10], which can ultimately impair insulin secretion. Age-related impairment of the intestinal barrier may increase exposure to bacterial products, thereby promoting activation of Toll-like receptor signaling pathways [17]. While we previously reported increased TLR4 ligands in pancreatic tissue [20], we here observed that TLR2 ligands were also elevated in old pancreatic tissue (Fig. 1A). To investigate the functional relevance of TLR2 signaling in the context of aging, young and old TLR2^−/−^ mice were compared with age-matched C57BL/6 (BL6) controls (Fig. 1B). In the glucose tolerance test (GTT), young TLR2^−/−^ mice displayed significantly lower blood glucose levels than young BL6 mice already 30 min after glucose injection (*p* = 0.0126). The mean blood glucose level also tended to be lower in old TLR2^−/−^ mice than in age-matched BL6 controls, although the difference did not reach statistical significance (*p* = 0.0590) (Fig. 1C). Fasting blood glucose levels were significantly reduced in old TLR2^−/−^ compared to their young TLR2^−/−^ control, but did not differ from age-matched BL6 mice (Fig. 1D). To assess insulin secretory capacity in more detail, Langerhans islets were isolated and stimulated with glucose ex vivo. As shown in Fig. 1E, under low glucose (LG, 1 mM glucose) stimulation, old BL6 mice showed lower secreted insulin compared to their young controls, whereas no differences were observed in TLR2^−/−^ islets. Under high glucose (HG, 25 mM glucose) stimulation, old TLR2^−/−^ mice secreted significantly more insulin compared to their young TLR2^−/−^ control and age-matched BL6 mice. Accordingly, the stimulation index (HG/LG) tended to be higher in young TLR2^−/−^ mice than in young BL6 controls, although the difference did not reach statistical significance (*p* = 0.0648). The index tended also to be higher in old than in young TLR2^−/−^ mice but this difference was likewise not statistically significant (*p* = 0.0939). No difference was observed between old TLR2^−/−^ and age-matched BL6 mice. Further, the total amount of secreted insulin during the assay (LG, HG and KCl stimulation) was significantly higher in old TLR2^−/−^ compared with age-matched BL6 mice, but also to their young TLR2^−/−^ controls. Collectively, these findings suggest that TLR2 contributes to the age-associated regulation of insulin secretion, as TLR2 deficiency enhanced selected measures of ex vivo insulin secretory capacity in aged islets without markedly altering in vivo glucose tolerance.

### Aging affects β-cell mass and islet composition, while the genetic loss of TLR2 attenuates age-related changes

**Fig. 2:**
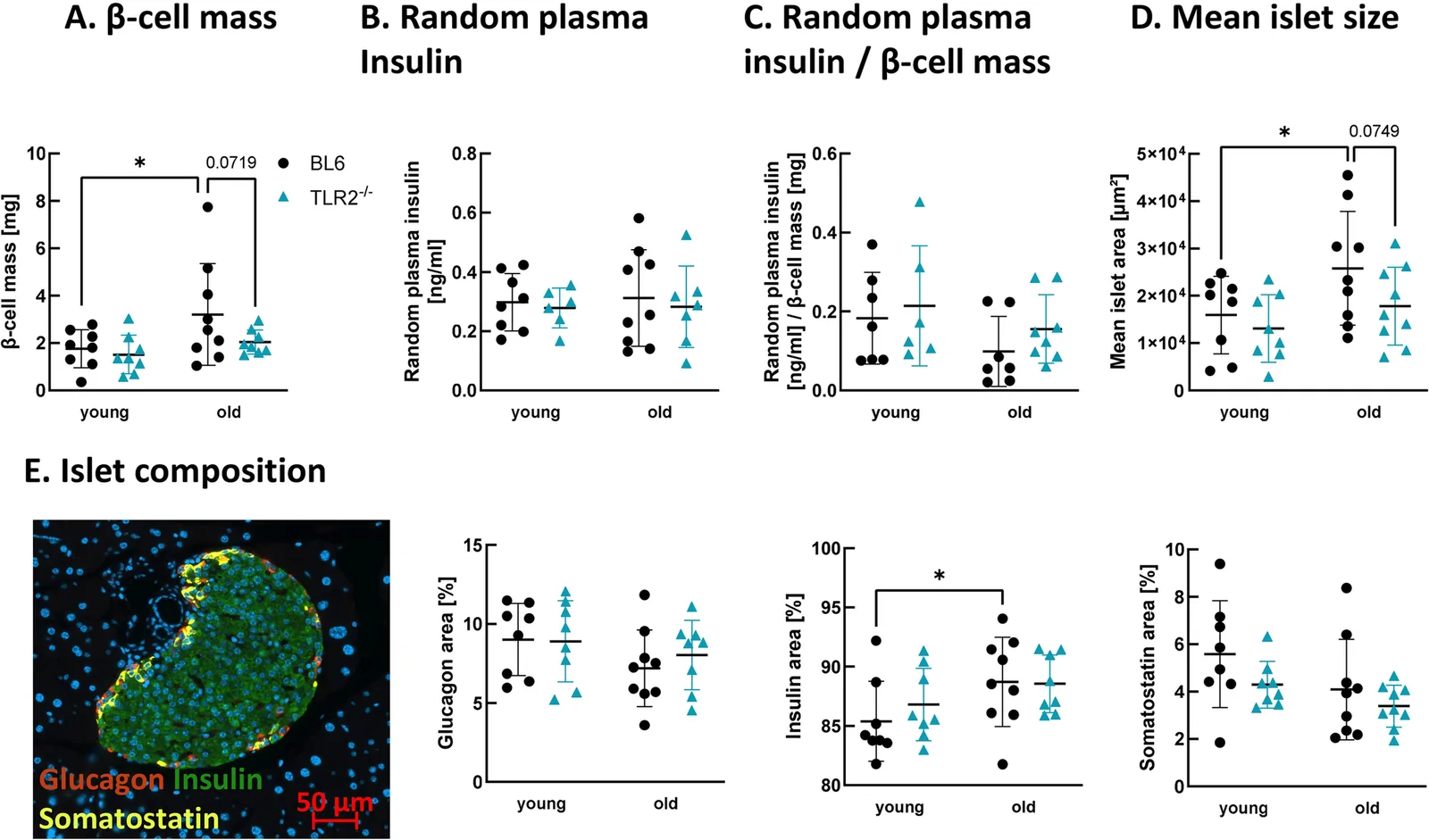
Effect of genetic deletion of TLR2 on β-cell mass, plasma insulin and islet composition in young and old mice. **A** Analysis of β-cell mass of pancreatic tissue. **B** Random plasma insulin. **C** Random plasma insulin normalized to β-cell mass. **D** Mean islet size assessed by islet composition. **E** Islets composition with a representative image and glucagon, insulin and somatostatin fractions in % to total islet area. All Data are represented as mean ± SD; *n* = 8–9; Two-Way ANOVA with Uncorrected Fisher’s LSD; *p < 0.05. TLR = toll-like receptor

Age-related alterations in β-cell mass and islet architecture have been interpreted as adaptive remodeling in response to metabolic demand and/or functional decline [12, 31]. While in our aging BL6 cohort a significant increase of β-cell mass could be observed, this was attenuated in old TLR2^−/−^ mice, which tended to have a lower β-cell mass compared to age-matched BL6 mice, although the difference did not reach statistical significance (*p* = 0.0719) (Fig. 2A). However, random plasma insulin levels were not changed, also the calculated ratio of plasma insulin per β-cell mass did not differ between groups (Fig. 2B, C). Mean islet area was also significantly increased in old BL6 mice, whereas old TLR2^−/−^ mice showed a trend toward smaller islets than age-matched BL6 mice, again without reaching statistical significance (*p* = 0.0749; Fig. 2D). When analyzing endocrine cell composition, insulin^+^ area fraction was higher in old BL6 mice compared with young BL6 controls, while endocrine cell area fractions (insulin^+^, glucagon^+^, and somatostatin^+^) remained unchanged with age in TLR2^−/−^ mice (Fig. 2E). Together, these findings suggest that TLR2 contributes to age-associated structural remodeling of pancreatic islets, whereas TLR2 deficiency attenuates several of these changes and is associated with partial preservation of structural features observed in young mice.

### Immune cell infiltration increases with age, but intra-islet immune cell accumulation is attenuated in old TLR2^−/−^ mice

**Fig. 3:**
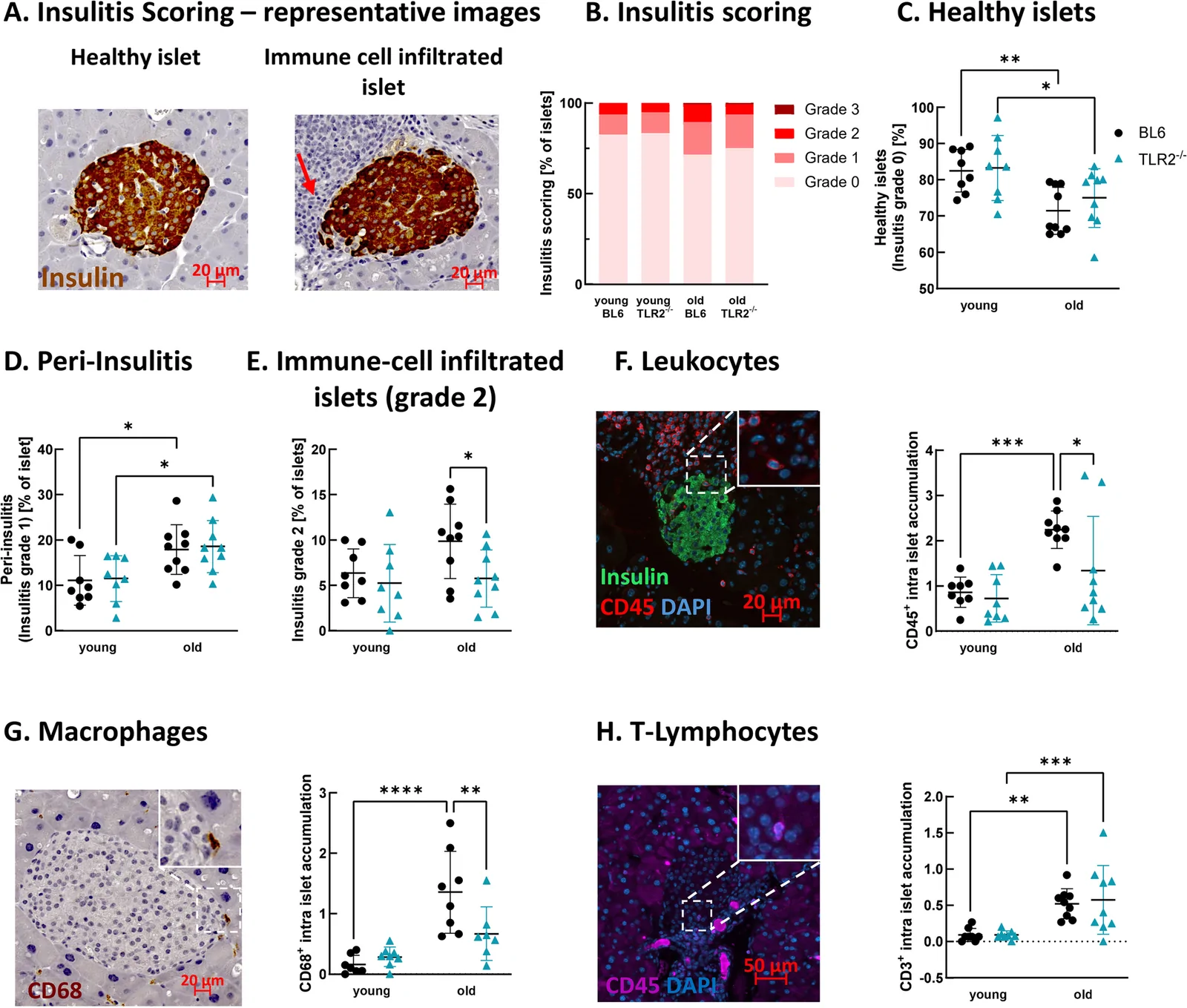
Effect of genetic deletion of TLR2 on insulitis and immune cell accumulation. **A** Representative images of insulitis showing a healthy islet and an infiltrated islet. **B** Distribution of insulitis grades in all samples. **C** Healthy, non-infiltrated islets (grade 0). **D.** Peri-Insulitis islets (grade 1). **E** Immune cell infiltrated islet (less than 50% immune cell infiltration, grade 2). **F** Representative image of CD45 staining and mean intra-islet leukocyte number (CD45^+^). **G.** Representative image of CD68 staining and mean intra-islet macrophage number (CD68^+^). **H** Representative image of CD3 staining and mean intra-islet T-lymphocyte number (CD3^+^). All Data are represented as mean ± SD or mean only (Insulitis scoring); *n* = 8–9; Two-Way ANOVA with Uncorrected Fisher’s LSD; *p < 0.05; **p < 0.01; ***p < 0.001; ****p < 0.0001. TLR = toll-like receptor

Cytokines released by immune cells can modulate the islet microenvironment by promoting immune cell recruitment and β-cell stress, but may also influence β-cell plasticity depending on the cytokine milieu. Figure 3A shows representative insulin-stained pancreatic sections illustrating a healthy, non-infiltrated islet (grade 0) and an islet with intra-islet immune-cell infiltration (grade 2). The overall distribution of insulitis grades showed an age-associated shift from healthy islets toward peri-insulitis and intra-islet infiltration, with a less pronounced grade 2 phenotype in old TLR2^−/−^ mice (Fig. 3B). With aging the proportion of healthy islets without immune cell accumulation decreased, in both BL6 and TLR2^−/−^ mice (Fig. 3C). Peri-insulitis, the immune cell accumulation around the islets increased with age in both genotypes (Fig. 3D). In contrast, grade 2 insulitis (intra-islet infiltration < 50%) was less frequent in old TLR2^−/−^ mice compared to age-matched BL6 mice (Fig. 3E). Additional immunofluorescence staining revealed that intra-islet leukocyte numbers (CD45^+^) increased with age in BL6 but not in TLR2^−/−^ mice (Fig. 3F). Among immune cell subtypes in particular macrophages (CD68^+^) increased with age in BL6 but not TLR2^−/−^ mice (Fig. 3G), while T-lymphocytes (CD3^+^) increased with age in both genotypes (Fig. 3H). Together, these findings suggest that aging promotes peri-insulitis and the loss of non-infiltrated islets in both genotypes, while TLR2 deficiency is associated with reduced age-related intra-islet leukocyte infiltration, particularly macrophage accumulation.

### LTA induces TLR2 and pro-inflammatory gene expression in old islets, which is attenuated by ortho-vanillin

**Fig. 4:**
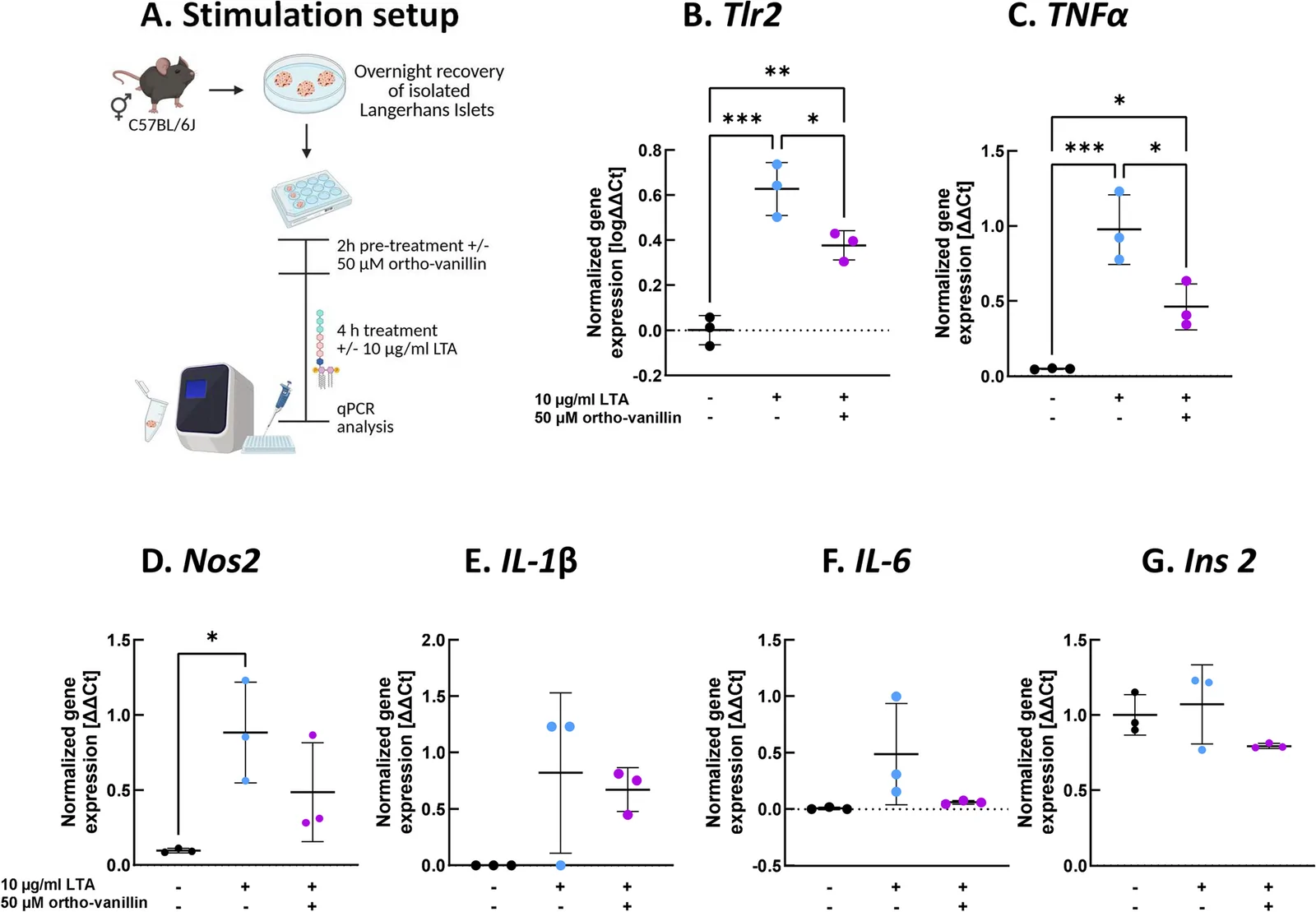
Lipoteichoic acid (LTA) and ortho-vanillin (oV) stimulation in old, murine Langerhans islets. **A** Experimental setup of Langerhans islet isolation and stimulation scheme; graphic created in BioRender. **B***Tlr2* gene expression after stimulation. **C***TNFα* gene expression after stimulation. **D***Nos2/iNOS* gene expression after stimulation. **E***IL-1β* gene expression after stimulation. **F***IL-6* gene expression after stimulation. **G***Ins2* gene expression after stimulation. All Data are represented as mean ± SD; *n* = 3; Ordinary One-Way ANOVA with Tukey’s multiple comparison test; *p < 0.05; **p < 0.01; ***p < 0.001. IL-1β = Interleukin-1 beta; Ins2 = Insulin 2; IL-6 = Interleukin-6; iNOS = inducible nitric oxide synthase. NOS2 = Nitric oxide synthase, inducible, TLR = toll-like receptor, TNFα = tumor necrosis factor alpha

Given that genetic deletion of TLR2 was associated with reduced intra-islet immune cell accumulation in vivo, we asked whether direct activation of TLR2 signaling in islets can induce cytokine expression and if this response is sensitive to pharmacological inhibition. Because it remains unclear to what extent Langerhans islets from aged mice respond to TLR2 ligands, Langerhans islets from old C57BL/6J mice were ex vivo stimulated with LTA with or without a 2-h pre-treatment with the TLR2 inhibitor ortho-vanillin (oV) (Fig. 4A). After 4 h LTA stimulation, *TLR2* gene expression was increased in the LTA treated group, and partially reduced by oV (Fig. 4B). This was accompanied by increased *TNFα* and *Nos2/iNOS* gene expression, which was likewise partially reduced by pre-treatment with oV (Fig. 4C, D). In contrast, *Il1β* and *Il6* expression did not change significantly (Fig. 4E, F), although variability increased following LTA stimulation. Besides that, no influence on *Ins2* gene expression was observed regardless of the treatment (Fig. 4G). Propidium iodide measurement of treated islets further showed no difference in islet viability after LTA or oV treatment, ensuring the observed readouts are not caused by treatment induced cell death (Supplementary Fig. S2A). Collectively, these findings show that LTA induced a selective pro-inflammatory transcriptional response in aged islets, which was partially attenuated by oV without affecting Ins2 expression or islet viability.

### Pharmacological inhibition of TLR2 in old mice attenuates immune cell accumulation and age-related β-cell mass increase

**Fig. 5:**
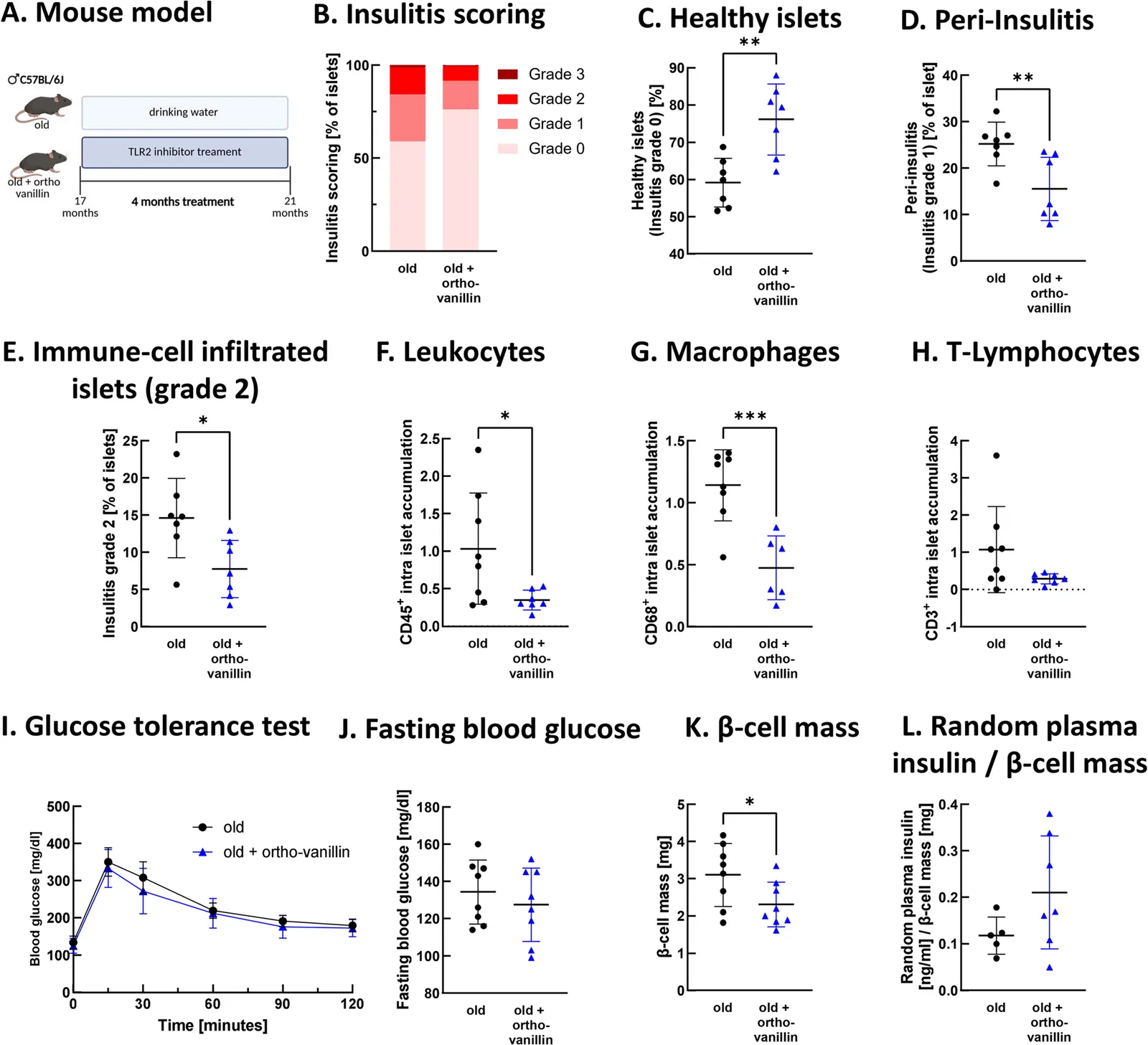
Effect of TLR2-inhibition in old mice: insulitis, immune cell accumulation and glucose tolerance. **A** Study design of the mouse experiment; graphic created in BioRender. **B** Distribution of insulitis grades. **C** Healthy, non-infiltrated islets (grade 0). **D** Peri-Insulitis islets (grade 1). **E** Immune cell infiltrated islet (less than 50% immune cell infiltration, grade 2). **F** Mean intra-islet leukocyte number (CD45^+^). **G** Mean intra-islet macrophage number (CD68^+^). **H** Mean intra-islet T-lymphocyte number (CD3^+^). **I** Glucose tolerance test. **J** Fasting blood glucose levels. **K** Analysis of β-cell mass of pancreatic tissue. **L** Random plasma insulin normalized to β-cell mass. All Data are represented as mean ± SD or mean only (Insulitis Score); *n* = 7–8; unpaired Student’s *t*-test or Two-Way ANOVA with Šídák’s multiple comparisons test (glucose tolerance); p * < 0.05; **p < 0.01; ***p < 0.001. TLR = Toll-like receptor

In a next step, male old C57BL/6J mice, were treated starting at 17 months of age, a time point at which bacterial products, including TLR2 ligands, are significantly elevated [21], using oV for 4 months with drinking water as vehicle control (Fig. 5A). Insulitis scoring revealed that oV treatment reduced immune cell accumulation in and around the islets (Fig. 5B). A higher proportion of healthy islets could be maintained with aging (Fig. 5C) and reduced frequencies of peri-insulitis and grade 2 infiltrated islets were observed due to TLR2 inhibitor treatment (Fig. 5D, E). Consistent with the scoring data, intra islet leukocytes were reduced in oV-treated mice (Fig. 5F). Immune cell subtype analysis showed in particular reduced macrophage accumulation (Fig. 5G) while intra-islet T lymphocytes (CD3^+^) were low in oV-treated mice and more variable in untreated old controls (Fig. 5H). Despite these anti-inflammatory effects, glucose tolerance and fasted blood glucose were not changed between old oV-and control mice (Fig. 5I, J). However, age-related β-cell mass increase was significantly reduced in oV-treated mice, while random plasma insulin level per β-cell mass remained unchanged (Fig. 5K, L). Collectively, oV treatment attenuated peri- and intra-islet immune-cell accumulation, particularly macrophage accumulation, and reduced the age-associated increase in β-cell mass without altering glucose tolerance or fasting blood glucose.

### Islet fibrosis is reduced by pharmacological TLR2 inhibition in aging and is similarly attenuated in TLR2^−/−^ mice

**Fig. 6:**
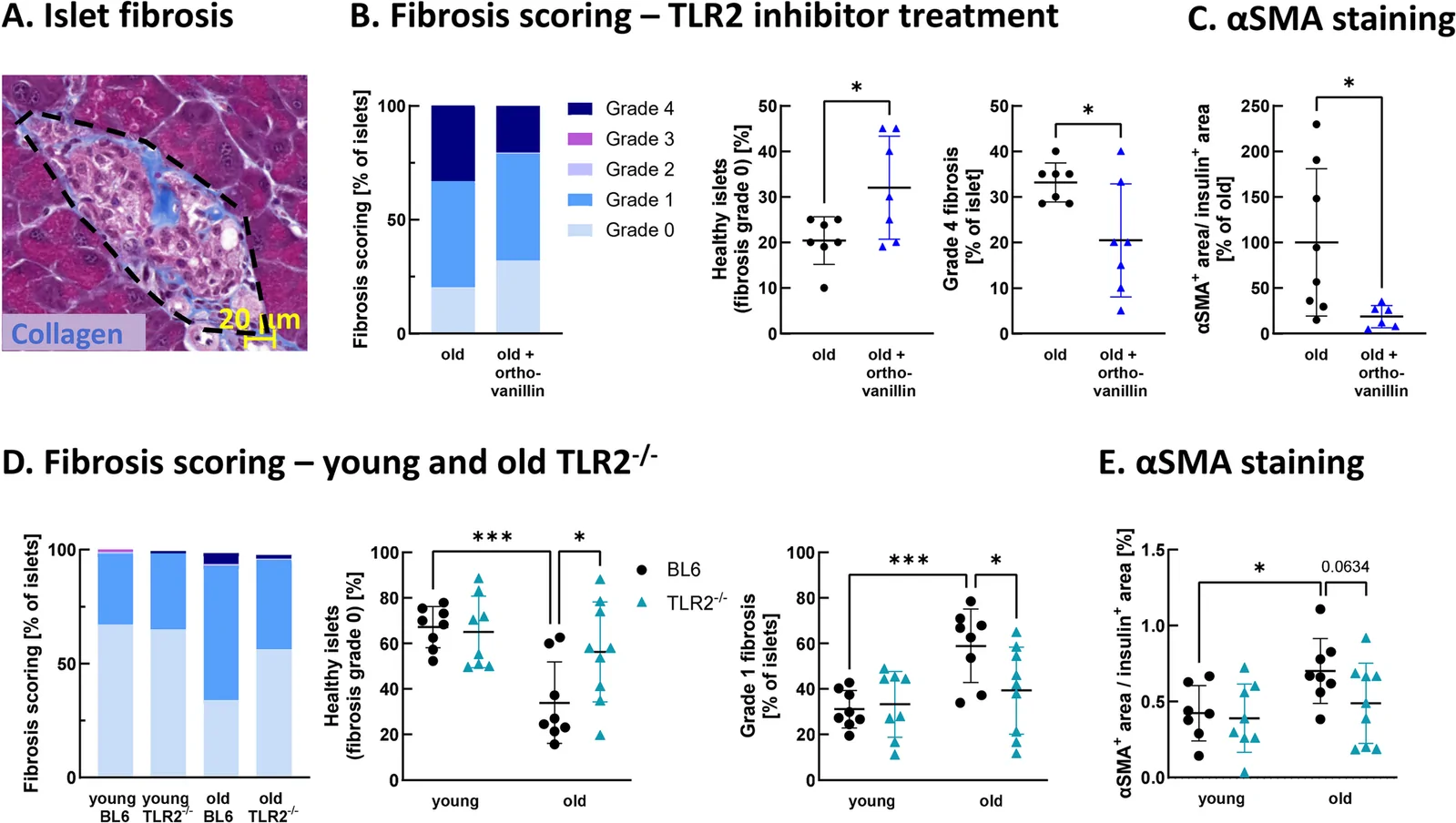
Langerhans islet fibrosis in aging is influenced by TLR2. **A** Representative image of a fibrotic islet. **B** Fibrosis Scoring of islets in old and old + ortho-vanillin treated mice, healthy non-fibrotic islets (grade 0) and fibrotic islets (grade 4); *n* = 7–8; unpaired Student’s *t*-test; *p < 0.05. **C** αSMA staining of islets to identify pancreatic stellate cells in old and old + ortho-vanillin treated mice, *n* = 7–8; unpaired Student’s *t*-test; *p < 0.05. **D** Fibrosis Scoring of islets in young and old TLR2^−/−^ mice and BL6 mice, healthy non-fibrotic islets (grade 0) and fibrotic islets (grade 1); *n* = 8–9; Two-Way ANOVA with Uncorrected Fisher’s LSD; *p < 0.05; **p < 0.01. **E** αSMA staining of islets in young and old TLR2^−/−^ mice and BL6 mice; *n* = 8–9; Two-Way ANOVA with Uncorrected Fisher’s LSD; *p < 0.05. All Data are represented as mean ± SD or mean only (Fibrosis Score)

An inflammatory environment, characterized by an increase in immune cells, also promotes structural remodeling by fibroblasts via cytokine crosstalk, leading to increased fibrosis with age [32]. Infiltrating and thickening extracellular matrix components such as collagen structures in islets as shown in Fig. 6A, are increased with age consistent with our previous observations [10]. oV treatment reduced fibrotic islets reflected by a higher proportion of non-fibrotic, healthy islets and a reduction in severely fibrotic grade 4 islets compared with untreated old controls (Fig. 6B). This was in line with a reduced activation of pancreatic stellate cells, as indicated by decreased αSMA staining in islets of oV-treated mice (Fig. 6C). Genetic deletion of TLR2 showed a comparable attenuation of age-associated fibrosis with a higher proportion of non-fibrotic, healthy islets, but in contrast especially mildly fibrotic grade 1 islets were reduced (Fig. 6D). Consistently, old TLR2^−/−^ mice showed a trend toward lower αSMA staining than age-matched BL6 mice, although the difference did not reach statistical significance (*p* = 0.0634). In contrast, αSMA staining was increased in old BL6 mice compared with young BL6 controls (Fig. 6E). Both pharmacological inhibition via oV treatment and genetic deletion of TLR2 attenuate age-associated islet fibrosis although the patterns of fibrosis reduction differed between the two models.

## Discussion

Updated concepts of the hallmarks of aging include chronic inflammation and dysbiosis, linking aging to increased exposure to microbial-derived signals [33, 34]. Elevated levels of bacterial products, potentially driven by age-related changes in microbiome composition but also disturbed intestinal barrier function, activate TLR signaling cascades, that induce inflammatory processes [17]. This inflammatory milieu contributes to multiple age-related diseases, including type 2 diabetes, in part by impairing β-cell function [3, 35]. Consistent with this concept, we recently showed, that pharmacological inhibition of TLR4 in old mice attenuates age-related changes of Langerhans islets [20]. Extending these findings, we now show that besides TLR4 ligands, TLR2 ligands also accumulate in old pancreatic tissue (Fig. 1A). TLR2 recognizes a wide range of damage- and pathogen-associated molecular patterns including LTA, triacetylated lipopeptides and peptidoglycans [23, 36]. One of the major mediators of TLR signaling are immune cells, particularly macrophages, which have been shown to contribute to islet inflammation via cytokine expression upon TLR2 activation [37, 38]. Ex vivo LTA stimulation of islets from old mice showed increased TLR2, Tnfα and Nos2/iNOS expression (Fig. 4 B-D). Given that this stimulation was performed on intact islets, the response is consistent with a major contribution from islet-resident immune cells within the islet microenvironment and was attenuated by pharmacological TLR2 inhibition with oV, supporting a TLR2-dependent inflammatory activation. Further, although short-term TLR2 stimulation of islets did not alter insulin gene expression (Fig. 4G), insulin expression may be affected over the long term under chronic low-grade inflammation.

Previous studies have shown, that genetic whole-body deletion of TLR2 improves glucose tolerance, insulin sensitivity and may protect against insulin resistance under metabolic challenges such as high-fat diet [39–41], supporting a role for TLR2 in obesity-associated inflammation [41]. Since aging is likewise characterized by increased low-grade inflammation [42], we assessed metabolic phenotypes in young and old mice. At 20 months of age, BL6 control mice still displayed preserved glucose tolerance compared to young controls, indicating maintained metabolic homeostasis at this age (Fig. 1C). Nevertheless, TLR2^−/−^ mice showed lower glucose excursions after glucose injection and reduced fasting glucose in old animals (Fig. 1C, D). In addition, islets from TLR2^−/−^ mice exhibited improved GSIS (higher stimulation index in young mice and increased insulin secretion in old mice; Fig. 1E), suggesting enhanced β-cell function due to TLR2 deletion. Further, age-related β-cell mass expansion is thought to precede overt glucose intolerance as a compensatory mechanism to sustain glucose homeostasis [31, 43]. Accordingly, old BL6 mice displayed a β-cell mass increase compared to their young control, accompanied by increased islet size and insulin positive area leading to altered islet composition (Fig. 2A, D, E). In contrast, old TLR2^−/−^ mice resembled their young control and showed a trend toward lower β-cell mass than aged-matched BL6 mice, although the difference did not reach statistical significance (*p* = 0.0719; Fig. 2A). Proliferating cell nuclear antigen (PCNA)-positive β-cell proliferation did not differ between age-matched groups (Supplementary Fig. S3A). This suggests that the attenuated β-cell mass expansion was not accompanied by detectable changes in proliferative activity at the investigated time point. However, differences in apoptosis or cumulative β-cell turnover cannot be excluded. Together with the improved GSIS phenotype, this suggests, that genetic deletion of TLR2 lowers the requirement for β-cell mass expansion during aging, potentially by reducing inflammatory pressure and/or improving secretory efficiency. Interestingly, pharmacological inhibition of TLR2 in old mice did not affect glucose tolerance, yet reduced the age-associated increase in β-cell mass with age (Fig. 5I-K) without altering PCNA-positive β-cell proliferation (Supplementary Fig. S3B), supporting a role of TLR2 signaling in driving age-related compensatory β-cell mass adaptation, while whole-body glucose handling remains largely unchanged under the conditions tested.

Studies suggest, that immune cells, particularly macrophages as part of the islet microenvironment, play a key role in regulation of islet homeostasis, adaption and β-cell function [44–48]. We and others have shown that islets accumulate immune cells with age [10, 13], a phenotype also referred to as insulitis and first described in type 1 diabetes [49–51]. This age-associated shift in the distribution of insulitis grades was partially modulated by TLR2 deficiency (Fig. 3B). In the genetic model, leukocyte accumulation was reduced in old TLR2^−/−^ mice compared with age-matched BL6 controls (Fig. 3F), consistent with a lower fraction of grade 2 insulitis islets (Fig. 3E), while percentages of healthy, non-infiltrated islets and peri-insulitis islets were not changed (Fig. 3C, D). In the pharmacological setting, old C57BL/6J mice treated with the TLR2 inhibitor showed reduced leukocyte accumulation compared with aged controls (Fig. 5F). In this cohort (21-month-old), TLR2 inhibition also shifted the distribution of insulitis stages towards more healthy, non-infiltrated islets and less peri-insulitis (Fig. 5C, D), alongside a reduced fraction of grade 2 insulitis islets (Fig. 5E). Importantly, the overall insulitis phenotype observed in 20-month-old BL6 mice in this study was less pronounced than we previously reported in 26-month-old mice [10], consistent with immune cell infiltration being a progressive, age-dependent process. Still, intra-islet macrophages, often considered as early initiator of insulitis that attract other immune cell subtypes via cytokine production [52], were less present in old TLR2^−/−^ and after TLR2 inhibition (Figs. 3G and 5G). This supports the notion, that TLR2 activation contributes to macrophage recruitment and polarization, as shown in other studies [53–55]. In contrast, T-lymphocyte accumulation in 20-month-old BL6 controls was rare, so we did not observe TLR2-dependent differences in the knockout mice (Fig. 3H). When we compared old TLR2 inhibitor-treated mice to their control, we observed a higher variability in T-lymphocyte number in the islets of old, untreated mice but again no significant changes compared to TLR2 inhibitor treated mice (Fig. 5H). Since at this stage of age T-lymphocytes are rarely present, TLR2-dependet effects may become more apparent at higher age, when T-lymphocytes are significantly increased in islets [10, 13]. However, changes in immune cell compartments could not be determined either at the islet level or systemically, limiting conclusions regarding the role of specific immune cell subtype changes. Beyond immune cell accumulation, age-related inflammation is closely linked to senescence and fibrotic remodeling. Senescent cells can amplify inflammation via senescence-associated secretory phenotype (SASP) factors and have been associated with elevated TLR2 expression, accordingly TLR2 blockade (e.g., oV) has been reported to reduce senescent cell burden and SASP factors [56–58]. In a parallel analysis performed in our collaboration, using liver tissue from the same treatment cohort as in the present study, oV treatment reduced senescence markers [21], further supporting a link between TLR2 signaling and senescence-associated tissue changes. However, since oV might act over TLR2-independent pathways and possesses anti-inflammatory and senolytic properties, further experiments with a structural distinct TLR2-inhibitor are needed.

Fibrosis represents another key feature of aging across various tissues, and TLR-driven pathways may contribute to fibrotic remodeling [32, 59]. In the pancreas especially quiescent stellate cell can be activated by cytokine stimulation and promote islet fibrosis by producing collagen [60, 61]. Both genetic TLR2 deletion and pharmacological TLR2 inhibition reduced islet fibrosis, which was accompanied by reduced αSMA staining (Fig. 6). Similar anti-fibrotic effects of TLR2 blockade were also observed in other tissues and were associated with decreased cytokine expression and reduced inflammation [21, 62, 63].

While our study shows that TLR2 contributes to age-related structural changes, it cannot be concluded whether the observed effects were mediated systemically or were islet-intrinsic. Because the TLR2^−/−^ model represents a whole-body knockout, the individual contributions of β-cells, immune cells, and stromal cells cannot be distinguished. Accordingly, the present findings support a role at the level of the TLR2 axis but do not establish a strictly islet-intrinsic mechanism. In addition, inhibition of TLR2 downstream signaling could not be directly quantified in pancreatic islets from oV-treated mice. Moreover, as the TLR2^−/−^ and BL6 mice were neither littermate-controlled nor co-housed, microbiota-related contributions to the observed phenotype cannot be excluded. Since oV may also exert TLR2-independent anti-inflammatory or senescence-modulating effects, cell-specific TLR2 deletion in islet-cell types and a structurally distinct TLR2 inhibitor are needed to unravel islet-intrinsic mechanisms. In addition, the study is limited by examining only male mice. Since islet-specific sex differences are already well documented [64–66], studies in female mice are necessary to determine whether these findings are sex-specific or broadly generalizable.

## Conclusion

In conclusion, our data show that targeting TLR2 attenuates age-associated changes in Langerhans islets in mice. Both pharmacological TLR2 inhibition and genetic TLR2 deletion reduced intra-islet immune cell accumulation and associated fibrotic remodeling. Furthermore, islets from TLR2^−/−^ mice exhibited enhanced insulin secretion, which may contribute to improved glucose regulation. However, at the time point investigated, old animals still displayed normal glucose tolerance, likely due to compensatory increases of β-cell mass, therefore, more pronounced systemic metabolic effects may only become detectable at later stages of aging.

## Supplementary Information

Supplementary Material 1.

Supplementary Material 2.

## Data Availability

The datasets used and/or analyzed during the current study are available from the corresponding author on reasonable request.

## Abbreviations

αSMA: Alpha smooth muscle actin
BL6: Control cohort of C57BL/6J mice for TLR2^−/−^ mice
BSA: Bovine serum albumin
BW: Body weight
DIfE: German Institute of Human Nutrition
GSIS: Glucose-stimulated insulin secretion
GTT: Glucose tolerance test
HG: High glucose
HPRT: Hypoxanthine-guanine phosphoribosyl transferase
i.p.: Intraperitoneal
IL-1β: Interleukin 1 beta
IL: Interleukin 6
iNOS: Inducible nitric oxide synthase
Ins 2: Insulin 2
KCI: Potassium chloride
KRBH: Krebs-Ringer bicarbonate HEPES buffer
LG: Low glucose
LTA: Lipoteichoic acid
NOS2: Nitric oxide synthase, inducible
oV: Ortho-vanillin
PBS: Phosphate buffered saline
PCNA: Proliferating cell nuclear antigen
RT: Room temperature
TLR: Toll-like receptor
TNFa: Tumor necrosisfactor alpha
