# Supplementary material for: Toll-like receptor 2 modulates age-associated insulitis and fibrotic remodeling of Langerhans islets
Source: Immun Ageing. 2026 Sep 12;23:32. doi: 10.1186/s12979-026-00598-0 (PMC13570508; doi:10.1186/s12979-026-00598-0)
Supplement: Supplementary file 1 [file 12979_2026_598_MOESM1_ESM.docx]

**Supplementary figure legends**

**Supplementary Fig. S1. Insulitis and Fibrosis Score. A.** Representative images of insulitis score grade 0 – grade 3, IHC staining of insulin, dotted line indicating the islet area, arrows indicating immune cells. **B.** Quantitative criteria for the insulitis score. **C.** Representative images of fibrosis score grade 0 – grade 4, Masson’s Trichrome staining, blue staining indicates collagen. **D.** Quantitative criteria for the fibrosis score. IHC = Immunohistochemistry

**Supplementary Fig. S2. Viability of LTA and oV treated islets. A.** Viability of islets after 4 h LTA or 6 h oV treatment. Isolated islets from aged C57BL/6J mice were overnight recovered and subsequently prepared for the stimulation with LTA [10 µg/ml], oV [50 µM] or control in RPMI^+^. The islets from two mice were pooled and then divided amongst the three test conditions. Biological triplicates were carried out. oV treated islets were incubated for 6 h while LTA was added 2h afterwards for 4h to mimic the experimental conditions of oV pre-treatment. Subsequently islets were picked, centrifuged [300 x G, 5 min, 4°C) and separated to a single cell suspension via 500 µl Accutase Cell Detachment Solution (PAN-Biotech, Germany) for 18 min at RT. After washing with RPMI^+^, single cells were resuspended in 100 µl RPMI^+^. 1µl propidium Iodide Solution (MiltenyiBiotec, Germany) was added and cells were directly measured the MACSQuant Analyzer 10 (MiltenyiBiotec) in the B3 channel. **B.** Gating strategy to identify alive and dead cells via propidium iodide solution. Data are mean ± SD, n =3, ordinary One-Way ANOVA with Tukey’s multiple comparison test. LTA = lipoteichoic acid, oV = ortho-vanillin.

**Supplementary Fig. S3. PCNA staining in islets to evaluate β-cell proliferation. A.** Representative image of PCNA staining (#2586, Cell Signaling, Germany) with insulin co-staining. PCNA-positive nuclei and total nuclei in the islet were counted threshold based in Zen3.5. **B.** β-cell proliferation measured by PCNA-positive nuclei in the islets of young and old TLR2^-/-^ mice and their BL6 control, n = 8-9; Two-Way ANOVA with Uncorrected Fisher's LSD. **C.** β-cell proliferation measured by PCNA-positive nuclei in the islets of TLR2-inhibitor treated mice and their respective controls, n = 7-8; unpaired Student’s *t*-test**.** All data are mean ± SD. PCNA = proliferating cell nuclear antigen, TLR = toll-like receptor
