# Supplementary material for: Toll-like receptor 2 modulates age-associated insulitis and fibrotic remodeling of Langerhans islets
Source: Immun Ageing. 2026 Sep 12;23:32. doi: 10.1186/s12979-026-00598-0 (PMC13570508; doi:10.1186/s12979-026-00598-0)
Supplement: Supplementary file 2 [file 12979_2026_598_MOESM2_ESM.docx]

**Supplementary tables**

Supplementary Table S1: Cut-off values for the image analysis

| Staining | Cut-off values for the analysis |
| --- | --- |
| Insulin | Zen 3.5: HLS color model  H: 0 - 44  L: 39 - 125  S: 33 - 171 |
| αSMA | fluorescence based  Zen 3.5: threshold between 756 and 5019 (scale of 0 – 16384)  Fiji ImageJ: pixel intensity min. 12 |
| CD45 | Zen 3.5: min. fluorescence intensity 800 (scale of 0 - 16384) |
| CD3 | Zen 3.5: min. fluorescence intensity 2000 (scale of 0 - 16384) |
| CD68 | Zen 3.5: clear brown chromogenic staining above background |
| PCNA | Zen 3.5: fluorescence-based thresholds (scale of 0 - 16384)  Islet: 2401 - 4654 (laser 488)  Nuclei: 520 - 7995 (laser 405)  PCNA-positive nuclei: 4675 - 9569 (laser 633) |

*Supplementary Table 2: Kappa Cohen’s scores and tables for the calculation of 200 random islets scored for insulitis or fibrosis by two independent investigators. Weighted calculations were performed with the online calculator in GraphPad https://www.graphpad.com/quickcalcs/kappa1/. The results for both scoring systems suggests a substantial agreement.*

| Kappa Cohen’s score | < 0: no agreement | between 0.00 and 0.20: slight agreement | between 0.21 and 0.40: fair agreement | between 0.41 and 0.60: moderate agreement | between 0.61 and 0. 80: substantial agreement | between 0.81 - 1.00: almost perfect agreement |
| --- | --- | --- | --- | --- | --- | --- |
| **Insulitis scoring** | | | | | | |
|  | **Grade 0** | **Grade 1** | **Grade 2** | **Grade 3** |  | **weighted Kappa** |
| **Grade 0** | 114 | 10 | 6 | 0 |  | **0.678** |
| **Grade 1** | 5 | 23 | 15 | 0 |  | **substantial agreement** |
| **Grade 2** | 2 | 4 | 19 | 0 |  |  |
| **Grade 3** | 0 | 0 | 0 | 2 |  |  |
| **Fibrosis Scoring** | | | | | | |
|  | **Grade 0** | **Grade 1** | **Grade 2** | **Grade 3** | **Grade 4** | **weighted Kappa** |
| **Grade 0** | 57 | 33 | 0 | 0 | 1 | **0.607** |
| **Grade 1** | 4 | 84 | 0 | 0 | 2 | **substantial agreement** |
| **Grade 2** | 0 | 2 | 2 | 0 | 1 |  |
| **Grade 3** | 0 | 1 | 0 | 1 | 0 |  |
| **Grade 4** | 0 | 5 | 0 | 0 | 7 |  |
